# The Efficacy of MRI in the diagnostic workup of cystic fibrosis-associated liver disease: A clinical observational cohort study

**DOI:** 10.1007/s00330-018-5650-5

**Published:** 2018-07-27

**Authors:** Sarah Poetter-Lang, Katharina Staufer, Pascal Baltzer, Dietmar Tamandl, Dina Muin, Nina Bastati, Emina Halilbasic, Jacqueline C. Hodge, Michael Trauner, Lili Kazemi-Shirazi, Ahmed Ba-Ssalamah

**Affiliations:** 1 General Hospital of Vienna (AKH), Department of Biomedical Imaging and Image-guided Therapy, Medical University Vienna, Waehringer Guertel 18-20, A-1090 Vienna, Austria; 2 Division of Gastroenterology and Hepatology, Department of Internal Medicine III, General Hospital of Vienna (AKH), Medical University of Vienna, Vienna, Austria

**Keywords:** Cystic fibrosis, Magnetic resonance imaging, Chemical shift imaging, Gallbladder, Liver diseases

## Abstract

**Purpose:**

To identify independent imaging features and establish a diagnostic algorithm for diagnosis of cystic fibrosis (CF)-associated liver disease (CFLD) in CF patients compared to controls using gadoxetic acid-enhanced MRI.

**Methods:**

A total of 90 adult patients were enrolled: 50 with CF, 40 controls. The CF group was composed of two subgroups: a retrospective test subgroup (n = 33) and a prospective validation subgroup (n = 17). Controls (patients with normal liver enzymes and only benign focal liver lesions) were divided accordingly (27:13). MRI variables, including quantitative and qualitative parameters, were used to distinguish CFLD from controls using clinical symptoms, laboratory tests and Debray criteria. Disease severity was classified according to Child-Pugh and Albumin-Bilirubin (ALBI) scores. Fifteen qualitative single-lesion CF descriptors were defined. Two readers independently evaluated the images. Univariate statistical analysis was performed to obtain significant imaging features that differentiate CF patients from controls. Through multivariate analysis using chi-squared automatic interaction detector (CHAID) methodology the most important descriptors were identified. Diagnostic performance was assessed by receiver-operating characteristic (ROC) analysis.

**Results:**

Three independent imaging descriptors distinguished CFLD from controls: (1) presence of altered gallbladder morphology; (2) periportal tracking; and (3) periportal fat deposition. Prospective validation of the classification algorithm demonstrated a sensitivity of 94.1% and specificity of 84.6% for discriminating CFLD from controls. Disease severity was well associated with the imaging features.

**Conclusions:**

A short unenhanced MRI protocol can identify the three cardinal imaging features of CFLD. The hepatobiliary phase of gadoxetic acid-enhanced MRI can define CFLD progression.

**Key Points:**

*• Using a multivariate classification analysis, we identified three independent imaging features, altered gallbladder morphology (GBAM), periportal tracking (PPT) and periportal fat deposition (PPFD), that could diagnose CFLD with high sensitivity, 94.1 % (95% CI: 71.3–99.9) and moderate specificity, 84.6 % (95% CI: 54.6–98.1).*

*• Based upon the results of this study, gadoxetic acid-enhanced MRI with DWI is able to diagnose early-stage CFLD, as well as its progression.*

## Introduction

Cystic fibrosis (CF) is one of the most common, lethal, autosomal recessive diseases of the Caucasian population. CF may affect any mucous-dependent organ, primarily the respiratory and digestive tracts [1]. So-called cystic fibrosis-associated liver disease (CFLD) may progress to end-stage liver cirrhosis, requiring curative liver transplantation [2]. CFLD, one of the cholestatic liver diseases, has been observed in up to 35% of CF patients during long-term follow-up [3]. Due to lung transplantation and optimised medical care, the survival of CF patients has improved [4, 5], such that CFLD is now considered the third leading cause of death, after primary lung disease and post-transplantation complications [6]. Therefore, early diagnosis of CFLD is crucial as some centres may initiate UDCA treatment as early as possible to improve liver function [7]. Unfortunately, both clinical and biochemical findings have low sensitivity and specificity for CFLD [8]. Likewise, data on the features of early-stage CFLD are still in rudimentary development. Ultrasound (US) or computed tomography (CT) can depict only the morphologic features of CFLD at very advanced stages [9]. Conventional magnetic resonance imaging (MRI), including MR cholangiopancreaticography (MRCP), is useful for assessing the hepatobiliary complications of CF. In previous studies, a wide range of hepatobiliary manifestations from hepatomegaly and diffuse fatty liver infiltration to severe cirrhosis with portal hypertension have been described [10]. Furthermore, biliary manifestations including strictures of intra- and extrahepatic bile ducts [11], as well as altered gallbladder morphology, such as cholelithiasis, sludge, micro- and macro-gallbladder have also been reported [9]. More recently, MRI techniques have been introduced, such as diffusion-weighted imaging (DWI) and hepatobiliary gadoxetic acid-enhanced MRI, which are increasingly used in hepatobiliary imaging [12]. Gadoxetic acid-enhanced MRI detects focal liver lesions and also provides information about regional and global function of the hepatobiliary system [13]. DWI, because of its inherent ability to detect subtle changes in hepatobiliary tissue [14], would be expected to detect CFLD at very early stages.

Our study aim was to identify independent imaging features and establish a diagnostic algorithm for the diagnosis of CFLD as compared with a control group using gadoxetic acid-enhanced MRI.

### Patients and methods

**Fig. 1:**
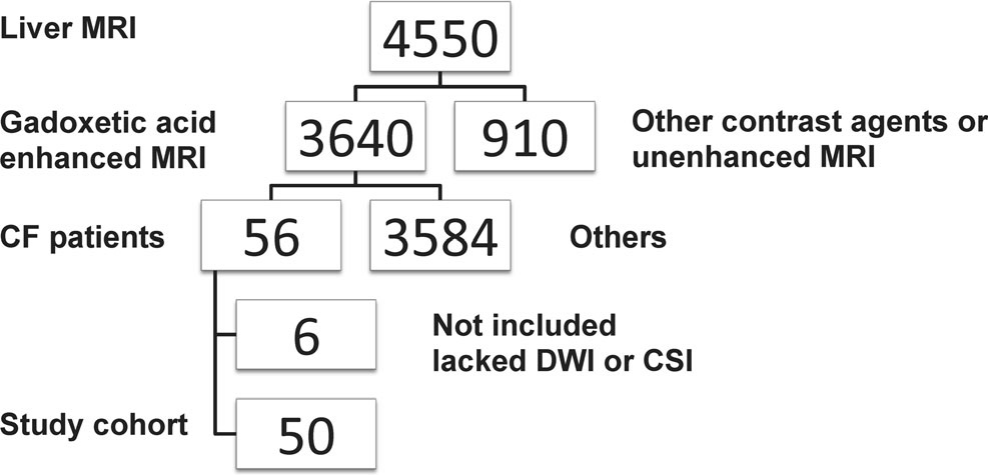
Flowchart: between 2011 and 2015, 3,640 patients underwent a standardized 3.0-Tesla gadoxetic acid-enhanced MRI of the liver. Fifty-six patients with cystic fibrosis (CF) were enrolled. Due to lack of diffusion-weighted imaging (DWI) or chemical shift imaging (CSI) we excluded six patients. Therefore, the final study cohort consisted of 50 patients

The ethics review board of our institution approved the prospective and retrospective data collection and analysis. However, informed consent was obtained only from the prospective group patients. The requirement for informed consent was waived for the retrospective group. The study cohort was determined from our institutional database as shown in the flow chart (Fig. 1).

A total of 90 adult patients were included, 50 with CF and 40 controls who were examined using identical parameters. From October 2011 to November 2013 we retrospectively enrolled 33 consecutive patients with CF, at any stage. We then gathered 27 consecutively liver-healthy controls who had an MRI for the diagnostic work-up of benign focal liver lesions. The controls had no further hepatobiliary abnormalities or elevated liver enzymes. These 60 patients from the retrospective CF group and controls were used as a training cohort.

From February 2014 to March 2015, we enrolled another 17 CF patients and 13 consecutive liver-healthy controls prospectively to validate the classification algorithm established with the training cohort. All control patients were gathered between March 2010 and December 2014. The retrospective and prospective CF groups were selected by the hepatologists after excluding other liver diseases.

They were sent to MRI due to inconclusive ultrasound screening. In all patients an identical gadoxetic acid-enhanced MRI protocol with DWI was applied. CFLD was defined by our hepatologists according to clinical symptoms, laboratory tests and Debray criteria [15].

In all patients, diagnosis of CF was definitively confirmed by our hepatologists. CF was diagnosed according to international criteria [16]. In addition, the majority of study patients had already had a double lung transplant for CF-associated lung disease at the time of study inclusion.

To estimate the severity of liver disease we used the Child-Pugh Score (CPS) for patients with cirrhosis [17], and the Albumin-Bilirubin (ALBI) score [18] for all patients.

Liver function tests, including serum bilirubin level, alkaline phosphatase (ALP), gamma-glutamyltransferase (GGT), aspartate aminotransferase (AST), alanine aminotransferase (ALT) and thrombocytes, were evaluated within 3 months of the MRI investigations. Clinical data for CF and controls were documented.

### MRI examination protocol

All MRI examinations were performed on a single 3-T MRI system (Magnetom TrioTim, Siemens Medical Systems, Erlangen, Germany). The MR imaging protocol is depicted on Table 1.

**Table 1:** MR protocol and examination parameters

Sequence	Section thickness (mm)	TR (ms)	TE (ms)	FOV (mm)	Phase direction	Flip angle	Acquisition time
GRE-T1 (flash 2D) in-phase	5	130	2.46	350	AP	70	2 × 17 s
GRE-T1 (flash 2D) opposed-phase	5	131	3.69	350	AP	70	2×17 s
T1 VIBE SPAIR axial, unenhanced, arterial, PV, transitional and hepatobiliary phases	1.7	2.67	0.97	430	AP	13	20 s
T1 VIBE SPAIR coronal PV phase and hepatobiliary phase	2	2.6	0.92	500	RL	13	20 s
T2 Haste coronal	4.5	805	76	450	RL	141	3×20 s
DWI axial TSE-EP	6	1700	73	380	AP	---	Resp. trigg. 4min.
T2 SE axial fs	5	2000	95	370	AP	165	Resp. trigg. 4 min
T2 Haste axial fs	5	1800	150	400	AP	150	3x20 s

*2D* two-dimensional, *AP* antero-posterior, *DWI* diffusion-weighted imaging, *FOV* field of view, *fs* fatsat, *GRE* gradient echo, *RL* right to left, *SPAIR* spectral-attenuated inversion recovery, *TE* echo time, *TR* repetition time, *TSE* turbo spin echo, *VIBE* volumetric interpolated breath-hold examination

All patients received a bolus injection of 0.025 mmol/kg/body weight gadoxetic acid (gadolinium ethoxybenzyl diethylenetriaminepentaacetic acid, Gd-EOB-DTPA, Primovist® Bayer) into a cubital or antecubital vein at 1 ml/s, followed by a 20-ml saline flush using a power injector.

### Image analysis

All MR images were reviewed on a commercial PACS system independently by two observers. Two radiologists, one with more than 20 years of experience in abdominal MR imaging (AB), the other in the sixth training year (SP), randomly evaluated the retrospective and prospective CF and control group patient images. Both observers were blinded to the subgroup, the pathological diagnosis and the clinical data. For training purposes, the readers jointly reviewed 20 sample MRI cases of patients with CF (n=10) and control patients (n=10) who were not included in this cohort study. The inter-observer variability was calculated.

Quantitative and qualitative assessment was performed.

### Quantitative assessment

Liver and spleen volumes were calculated by measuring the maximum dimension of the liver and spleen in three perpendicular axes: craniocaudal (CC), latero-lateral (LL) and antero-posterior (AP) [19]. In addition, the portal vein diameter was measured for each patient and a diameter above 12 mm was defined as abnormal [20].

The signal intensity (SI) in the left liver lobe, and in liver segments VI, VII and VIII were obtained and the relative liver enhancement (RLE) was calculated by measuring the SI in the left liver lobe and in segments VI, VII and VIII in unenhanced T1-weighted images, as well as the T1-weighted images, 20 min after the administration of gadoxetic acid in the HBP using the following formula:

$$ \mathrm{RLE}\ \left(\%\right)=\frac{\mathrm{HBP}\ \mathrm{enhanced}\ {\mathrm{SI}}_{\mathrm{liver}}-\mathrm{unenhanced}\ {\mathrm{SI}}_{\mathrm{liver}}}{\mathrm{unenhanced}\ {\mathrm{SI}}_{\mathrm{liver}}} $$

Furthermore, the SI of the spleen on in- and opposed-phase images was measured to calculate the corrected chemical shift imaging (CSI) hepatic fat fraction [21].

$$ {\mathrm{CSI}}_{\mathrm{hepatic}\ \mathrm{fat}\ \mathrm{fraction}\ \mathrm{spleen}\ \mathrm{correction}}=\frac{\left({\mathrm{SI}}_{\mathrm{in}\ \mathrm{phase}\ \mathrm{liver}}/{\mathrm{SI}}_{\mathrm{spleen}}\right)\hbox{--} \left({\mathrm{SI}}_{\mathrm{opposed}\ \mathrm{phase}\ \mathrm{liver}}/{\mathrm{SI}}_{\mathrm{spleen}}\right)}{2\mathrm{x}\ {\mathrm{SI}}_{\mathrm{in}\ \mathrm{phase}\ \mathrm{liver}}/{\mathrm{SI}}_{\mathrm{spleen}}} $$

All quantitative assessments were performed by both readers in consensus.

### Qualitative assessment

For the qualitative assessment, we scored the presence or absence of distinct qualitative MRI features, some taken from the literature [22, 23], as well as those from our longstanding clinical experience in MRI liver imaging.

A total of 15 qualitative features representing diffuse and focal liver changes on distinct MR-sequences were rated as present =1 or absent =0, including: 1 *–* diffuse steatosis, 2 *–* periportal fat deposition (PPFD) (a linear periportal SI decrease on opposed-phase compared to in- phase), 3 *–* periportal tracking (PPT) on DWI (a linear periportal increase in signal intensity), 4/5 *–* bile duct abnormalities (BDA) on T2-weighted/MRCP images and T1-weighted post-contrast images (irregularities of either stenosis or segmental dilatation or both), 6 /7 *–* heterogeneous liver parenchyma on DWI or and T1-weighted post contrast images, 8 *–* the degree of hepatobiliary uptake (this was evaluated both quantitatively by measuring the relative liver enhancement and qualitatively by visual impression in comparison to the kidney *–* brighter, equal or less bright), 9 *–* timely hepatobiliary excretion (presence of the contrast media in the common bile duct or even in the duodenum within 20 min after administration of contrast media (CM) was defined as timely excretion), 10 *–* altered gallbladder morphology (GBAM) (this includes micro-, macro-gallbladder, sludge and/or the presence of stones), 11 *–* periportal fibrosis (PPF) (means linear periportal decrease in signal intensity on DWI), 12 *–* the presence of regenerative nodular hyperplasia (RNH), 13 *–* widening of the fissures and hilum [24], 14 *–* portal vein dilatation (the portal vein diameter was measured on the pv post-contrast images), and 15 *–* the presence of lymph nodes in the hepatoduodenal ligament.

### Statistical analysis

Statistical analysis was performed using the IBM SPSS version 22.0 and the MedCalc statistical software version 15.4 for Windows.

Descriptive statistics were calculated to describe the study sample and were expressed as mean, range and absolute numbers. Categorical univariate variables (15 single-feature descriptors) were expressed as count and proportions and analysed using univariate Chi-square and Fisher’s exact tests. Count variables were analysed and compared between groups using non-parametric Whitney-Mann-U tests.

Levels of interobserver agreement were assessed using Cohen's kappa statistics, as defined in a study by Landis and Koch. Significant variables (*p* < 0.05) at univariate analysis were used as input variables for Bonferroni-corrected, ten-fold, cross-validated multivariate classification analysis (Chi-squared Automated Interaction Detection Algorithm - CHAID) [25]. The minimal case number for parent nodes was set to ten, for child nodes to five. All CHAID analyses were performed using standard proprietary SPSS procedures.

First, the results of the retrospective cohort were obtained and then tested for the prospective cohort. The diagnostic accuracy of the classification tree was evaluated by receiver-operating characteristics (ROC) curve (AUC) analysis with 95% confidence intervals (Cis) using Medcalc.

## Results

### Demographic data

A total of 90 adult patients were enrolled, 50 with CF and 40 without liver disease (controls). The retrospective (test) CF subgroup (n = 33, mean age 30.3 ± 9.7 years) consisted of 15 male and 18 female patients. The prospective (validation) group (n = 17, mean age 35.8 ± 11.2 years) consisted of nine male and eight female patients (Table 2).

**Table 2:** Patient characteristics and laboratory tests

	CF Retrospective group	CF Prospective group	Controls 1	Controls 2	*p*-value Retrospective group vs. controls 1	*p*-value Prospective group vs. controls 2
Patient number	33	17	27	13	n.s.	n.s.
Males	15	10	10	8	n.s.	n.s.
Females	18	7	17	5	n.s.	n.s.
Mean age (y)	30.3 ± 9.7	35.8 ± 11.2	51.2 ± 16.9	48.9 ± 13.9	<0.001	<0.001
Serum bilirubin (<1.2 mg/dl)	0.89±1.6	0,51±0.25	0.74±0.57	0.71±0.66	0.60	0.84
AP (35–105 U/L)	135.7±138.4	106.8±64.3	63.0±17.2	58.47±13.45	<0.05	<0.05
GGT (<40 U/L)	82.1±128.1	52.4±96.6	32.4±28.3	33.73±33.66	<0.05	<0.05
AST (<35 U/L)	39.2±61.4	30.5±16.6	30.3±31.5	33.95±39.17	0.47	0.52
ALT (<35 U/L)	41.8±50.9	37.9±35.0	26.7±15.4	27.57±17.14	0.11	0.07
Serum Albumin (35–52 g/L)	38.42±10.5	35.3±13.3	41.8±6.9	43.8±3.8	0.15	0.18

Data are presented as means ± standard deviations Laboratory tests: alkaline phosphatase (AP), gamma-glutamyltransferase (GGT), aspartate aminotransferase (AST), alanine aminotransferase (ALT) *p*-values are based upon Whitney-Mann-U tests *n.s.* not significant

### Clinical and laboratory parameters

Comparing the two CF subgroups to the controls, only the cholestatic parameters, i.e. ALP and GGT, were significantly higher in the CF groups (*p*< 0.05; CF vs. controls). The remaining liver function parameters were not significantly different between the two groups (Table 2).

From the 50 CF patients only eight patients showed liver cirrhosis according to the Child-Pugh score. There were three CPS A and three CPS B in the retrospective CF group and two CPS A in the prospective CF group. The remaining patients had no clinical evidence of liver cirrhosis. In the retrospective CF group, 25 patients were ALBI 1 and eight patients ALBI 2. In the prospective CF group, 13 patients had an ALBI 1 score, and four patients had an ALBI 2 score (Table 3).

**Table 3:** Association between severity of liver disease and Albumin-bilirubin (ALBI) score and Child-Pugh score (CPS) in the retrospective and prospective cystic fibrosis (CF) groups

	CF Retrospective group	CF Prospective group	Periportal fibrosis	Irregular liver margins	BDA in the HPB	Contrast media-uptake
ALBI	25 patients 1 8 patients 2	13 patients 1 4 patients 2	*p* = 0.37	*p* = 0.11	*p* = 0.47	*p* < 0.005
CPS	3 patients A 3 patients B	2 patients A	*p* < 0.005	*p* < 0.005	*p* < 0.005	*p* = 0.18

To estimate the severity of liver disease the Albumin-bilirubin (ALBI) score (A1=ALBI score grade 1=early stage, A2=ALBI score grade 2=moderate stage, A3=ALBI score grade 3=advanced stage, not available) and the Child-Pugh Score (CPS) for patients with cirrhosis were applied (A=well compensated cirrhosis, B=moderate functional compromised cirrhosis, C= decompensated cirrhosis, not available)

*p*-values are based upon Pearson chi-square tests

### Imaging parameters

#### Quantitative features univariate analysis

Our results showed that splenic volume was significantly higher in the two CF subgroups, as compared to the controls (*p*<0.05). The portal vein diameter (*p*=0.43) and liver volume (*p*=0.18) did not differ significantly. Likewise, the degree of hepatic steatosis (*p*=0.90) and the RLE (*p*=0.91) were not significantly different in either CF group compared to the controls (Table 4).

**Table 4:** The quantitative values of the cystic fibrosis (CF) groups and controls

Group	CF Retrospective group	CF Prospective group	Controls 1	Controls 2	*p*-value Retrospective group vs. Controls 1	*p*-value prospective group vs. controls 2
Patient number	33	17	27	13	n.s.	n.s.
Liver vol. median (cm^3^)	1,025 (805–1,174)	1,047 (921–1,325)	1,194 (823–1,364)	1,023 (724–1,221)	1	0.313
Spleen vol. median (cm^3^)	136 (111–179)	193 (115–259)	87 (60–121)	84 (67–107)	0.0008	0.002
Median portal vein diameter (mm) in post CE pv-images	11 (10–13)	13 (11–15)	11 (10–13)	10 (10–12)	0.887	0.061
RLE (%)	169 (140–197)	187 (130–208)	158 (140–182)	157 (135–197)	0.542	0.392
Liver fat fraction CSI (%)	4 (0–12)	5 (1–13)	1 (1–5)	3 (0–5)	0.138	0.302

Data are presented as median and interquartile range (IQR)

*Vol.* volume, *RLE* relative liver enhancement, *CE* contrast-enhanced, *CSI* chemical shift imaging, *pv* portal venous

*p*-values are based upon Mann-Whitney-U tests

The MR imaging features of cirrhosis including periportal fibrosis, irregular liver margins and bile duct abnormalities in the hepatobiliary phase associated well with CPS. The CM-uptake as a functional parameter associated significantly with the ALBI score, which, again, was indicative of advanced liver disease (Table 3).

#### Qualitative features univariate analysis

As there was a very high inter-reader agreement using Cohen’s kappa (κ= 0.8), the qualitative assessment of the MR images of the more experienced reader was taken for further analysis. These showed significantly more patients with several distinct MR features in both the retrospective and prospective CF groups compared to the controls *p* < 0.001 (Table 5), but there were no essential differences between the CF subgroups *p* > 0.05.

**Table 5:** The 15 qualitative features of cystic fibrosis (CF)

	CF Retrospective group	CF Prospective group	Controls 1	Controls 2	*p*-value Retrospective group vs. controls 1	*p*-value Prospective group vs. controls 2
Patient number	33	17	27	13	n.s.	n.s.
Diffuse fatty liver changes	9	8	4	4	0.119	0.25
Periportal fat deposition	18	11	4	0	<0.001	<0.001
Periportal tracking DWI	22	12	5	4	<0.001	0.002
Bile duct abnormalities MRCP T2w	12	2	0	0	0.002	0.002
Bile duct abnormalities T1w post CE	14	5	0	0	0.001	<0.001
Heterogeneous liver parenchyma DWI	16	5	0	0	<0.001	<0.001
Heterogeneous liver parenchyma T1w post CE	14	6	0	0	<0.001	<0.001
Degree of hepatobiliary contrast uptake normal	26	14	27	12	0.138	0.45
Timely hepatobiliary excretion	24	15	26	11	0.07	0.095
Gallbladder alterations	24	13	1	2	<0.001	0.013
Periportal fibrosis	10	1	0	0	0.003	0.007
RNH	4	0	0	0	0.075	0.074
Widening of the fissures and hilum	19	4	0	0	<0.001	<0.001
Portal vein diameter Post-CE pv-images >12 mm	11	8	8	3	0.138	0.058
Abnormal lymph nodes in the hepatoduodenal ligament	12	10	9	1	0.053	0.011

*DWI* diffusion-weighted images, *CE* contrast-enhanced, *RNH* regenerative nodular hyperplasia, *pv* portal venous

*p*-values are based upon Pearson chi-square or Fisher´s exact tests as appropriate

#### Qualitative features multivariate analysis

**Fig. 2:**
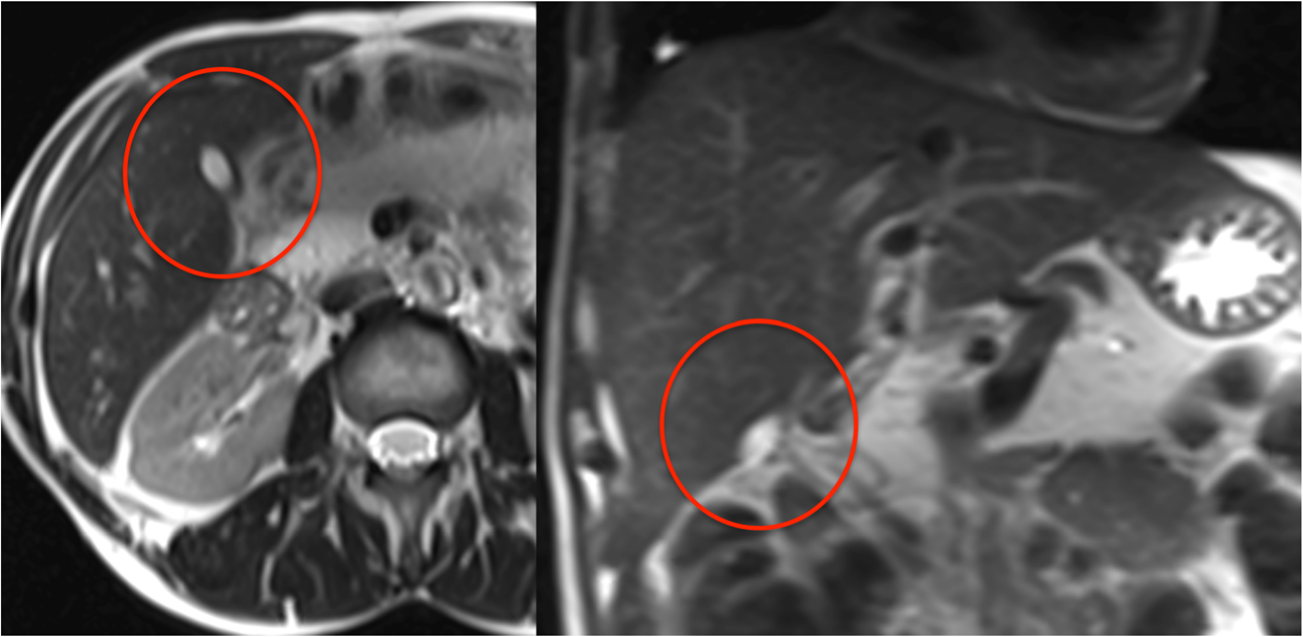
A 32-year-old male cystic fibrosis (CF) patient: axial and coronal T2-weighted images show an example of altered gallbladder morphology (GBAM), illustrated by a micro-gallbladder. The altered, distinctly shrunken gallbladder is well depicted on the T2-weighted axial and coronal images

**Fig. 3:**
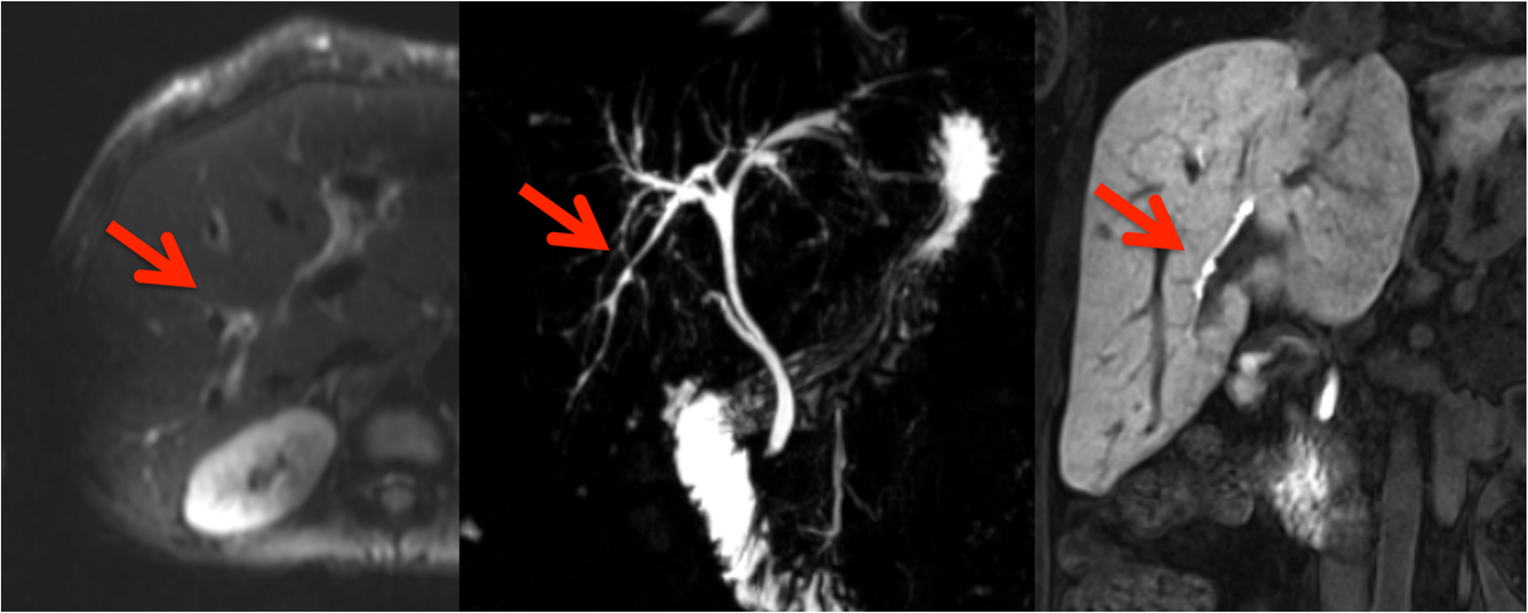
In this 25-year-old male cystic fibrosis (CF) patient periportal tracking (PPT) is shown on the diffusion-weighted images, demonstrating a band-like hyperintense signal alteration along the portal triad. Bile duct abnormalities (BDA) are shown in the T2-weighted magnetic resonance cholangiopancreaticography images and in T1-weighted images, in the hepatobiliary phase (20 min after the administration of gadoxetic acid) by contour irregularities of the intrahepatic bile ducts

**Fig. 4:**
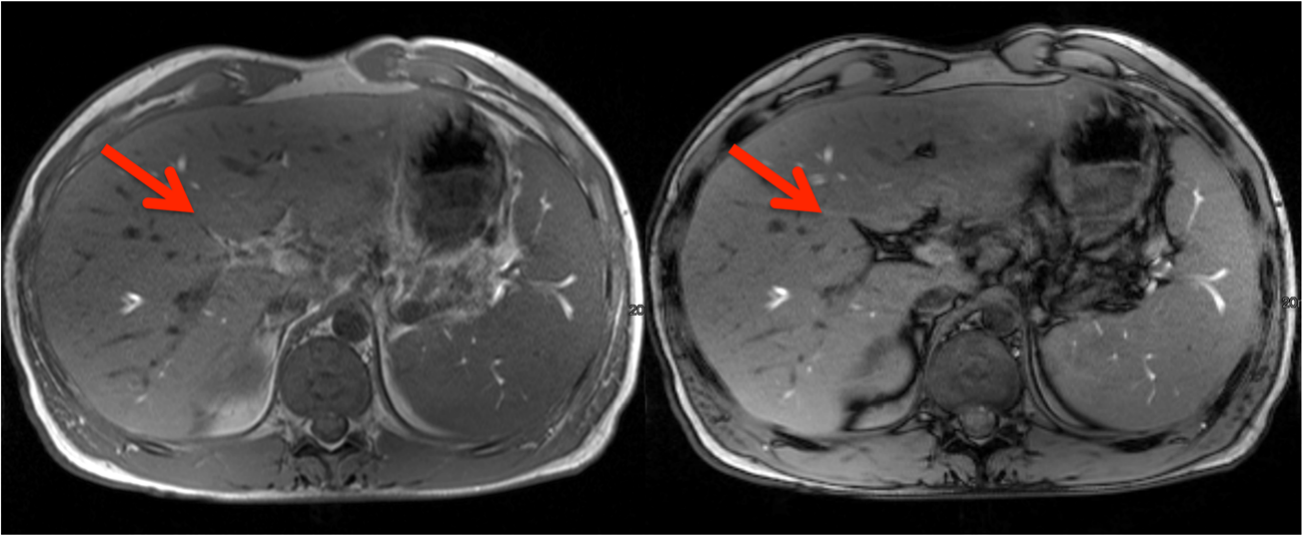
A 23-year-old male cystic fibrosis (CF) patient: T1-weighted in- and opposed-phase images demonstrate the periportal fat deposition (PPFD) as a band-like signal intensity loss on the opposed phase image compared to the in-phase image along the fissure of the porta hepatis

**Fig. 5:**
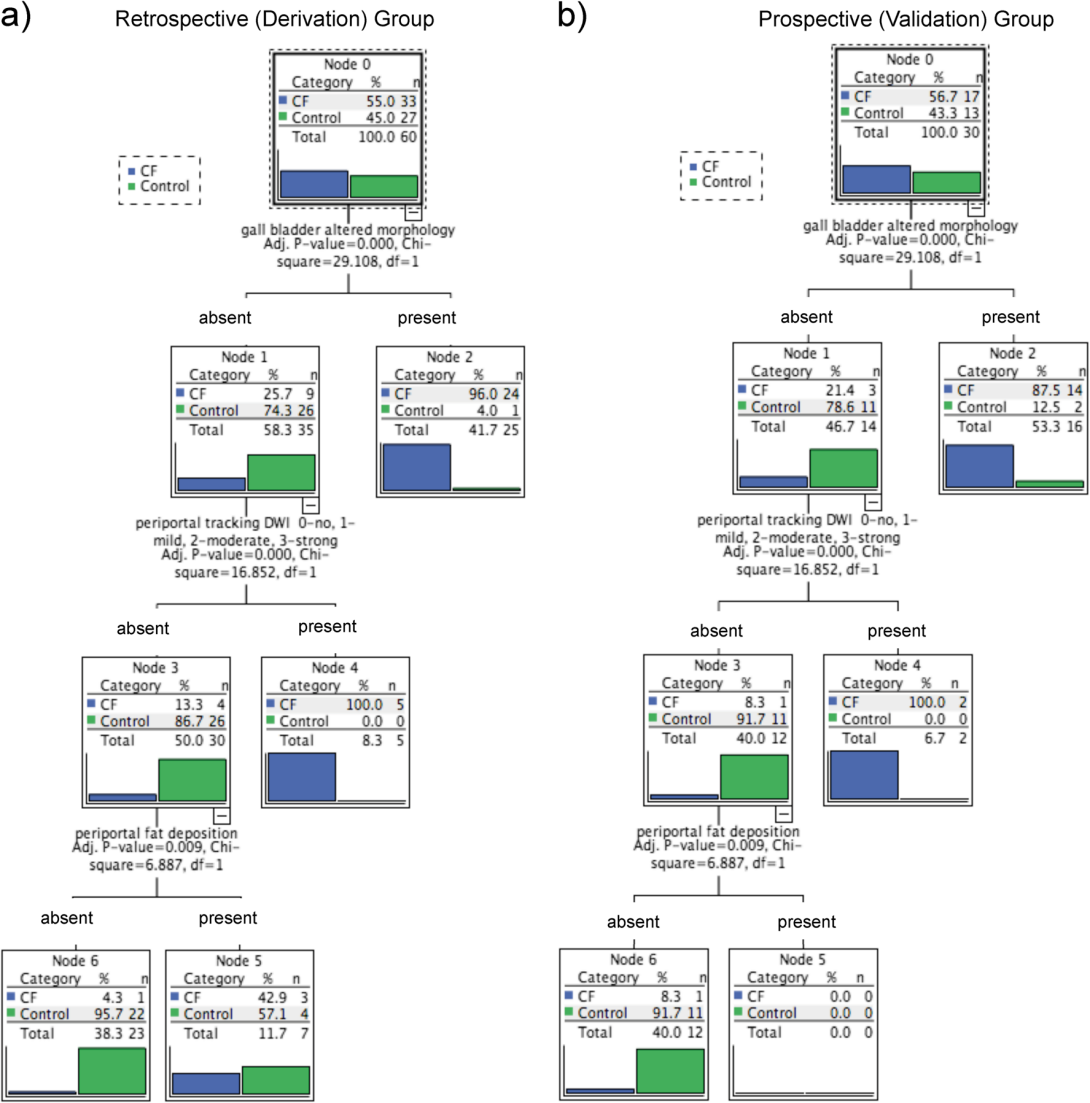
**(a**) The results, obtained from the multivariate (CHAID-Chi-Squared Automated Interaction Detection Algorithm) analysis for the retrospective cystic fibrosis (CF) group, show three independent MR imaging predictors of cystic fibrosis-associated liver disease (CFLD): the presence of altered gallbladder morphology (GBAM), periportal tracking (PPT) on diffusion-weighted imaging (DWI), and periportal fat deposition (PPFD) on chemical shift imaging (CSI) in a tree flow chart. (**b**) The tree flow chart for the prospective group confirmed the results obtained from the retrospective group

Among the classification features, the resulting CHAID tree flow chart for the retrospective CF group (Fig. 5) determined three imaging descriptors that had highly statistically significant differences between CFLD patients and controls, namely: (1) the presence of altered gallbladder morphology (GBAM) (Fig. 2); (2) periportal tracking (PPT) seen on DWI (Fig. 3); and (3) periportal fat deposition (PPFD) seen on T1 chemical shift imaging (Fig. 4). Furthermore, GBAM was the initial splitting predictor, separating those with a high probability of CFLD from the control group (*p*= 0.001).

For the retrospective group, GBAM had a 96% PPV for CFLD. The PPV was 100% in those CF patients who had normal gallbladder morphology but PPT. The NPV was 96% for CFLD in those patients who did not have GBAM, PPT or PPFD. These three imaging predictors had a sensitivity of 97.0 % (95% CI: 84.2*–*99.9) and a specificity of 81.5 % (95% CI: 61.9*–*93.7) for the discrimination of CFLD from controls.

The prospective CF group confirmed the results of the retrospective CF group (Fig. 5). Likewise, GBAM was also the initial splitting predictor in the validation group, according to the CHAID tree flow chart. Fourteen of 17 CF patients had evidence of GBAM, as opposed to two of 13 in the control group (*p* = 0.0001). Two-thirds of CF patients with normal gallbladder morphology showed PPT whereas no control patients did. For the prospective/validation group, the PPV of GBAM for CFLD was 88%. Furthermore, the PPV was 100% if gallbladder morphology was normal but there were signs of PPT. The NPV was 92% for the diagnosis of CFLD if patients did not have GBAM, PPT or PPFD. In the validation group, these three imaging predictors had a sensitivity of 94.1 % (95% CI: 71.3*–*99.9) and a specificity of 84.6 % (95% CI: 54.6*–*98.1) for the discrimination of CFLD from normal patients.

**Fig. 6:**
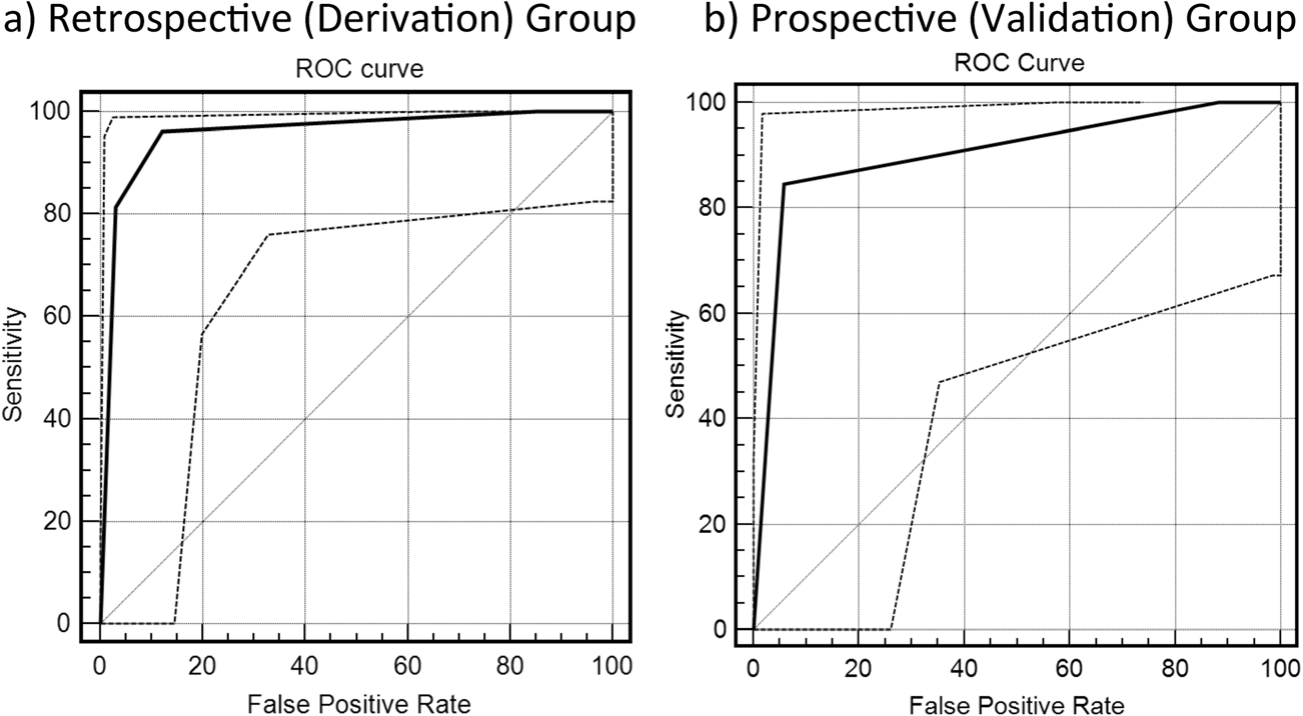
The area under the receiver-operating characteristic (ROC) curve used to evaluate the diagnostic efficacy in distinguishing cystic fibrosis-associated liver disease (CFLD) from the control group is 0.96 for the retrospective cystic fibrosis (CF) group (**a**) and 0.90 for the prospective/validation CF group (**b**)

Furthermore, the area under the ROC curves used to evaluate the diagnostic efficacy in distinguishing CFLD from the controls was 0.96 and 0.90 (Fig. 6) for the test and validation groups, respectively.

## Discussion

Our results obtained from the multivariate (CHAID) analysis showed that the most important and independent MR imaging predictors of CFLD are: (1) the presence of altered gallbladder morphology (GBAM); (2) periportal tracking (PPT) on DWI; and (3) periportal fat deposition (PPFD) on CSI. Using the CHAID statistical method, these three features, i.e. 1*–*3, also cited in previous studies [26–29], emphasise the fact that CFLD belongs to the group of cholestatic liver diseases [30]. Furthermore, the results from the retrospective cohort were validated by the prospective CF group.

GBAM, as described in the classic cholestatic liver disease PSC, turned out to be the highest-order discriminator between patients with CFLD and controls [32, 33].

In CFLD, the GB alterations have been considered to be due to defects in gallbladder motility and emptying [26, 27, 31]. We assume that CFLD patients have dysfunctional mucosa, which causes these changes either due to increased bile production and decreased biliary hydrophobicity or disrupted enterohepatic circulation of bile acids [31, 32].

The second-order predictors were PPT on DWI and the third PPFD on chemical shift imaging, respectively. Ductopenia, due to inflammation and oedema along the bile ducts or portal triad, results in wall thickening and fibrosis, which leads to vanishing duct syndrome, a pathognomonic feature of cholestatic liver diseases [33, 34]. Indeed, correlation of US with MR findings showed that periportal echogenicity is more often due to fat rather than fibrosis, as our findings confirmed (i.e. PPFD) [35]. Periportal tracking and periportal fat deposition are probably caused by geographic inflammation and oedema along the portal triad, again, characteristic for cholestatic liver diseases. As in PSC, the literature describes a range of intra- and extrahepatic biliary abnormalities for CFLD, including sludge, cholelithiasis, strictures, with or without cholangitis, and periductal fibrosis [36]. Although our results showed a high sensitivity (94.1%), the specificity was rather moderate (84.6 %), likely due to the fact that sludge and gallstones are so non-specific, and are also likely to be found in asymptomatic controls. Even by entering the statistically significant laboratory markers from the univariate analysis (ALP and GGT) to the multivariate analysis, there was no incremental value with regard to sensitivity and specificity.

However, in clinical practice, the presence of these imaging features (GBAM, PPT and PPFD), in combination with elevated ALP and GGT is extremely rare in healthy patients and highly suggestive of CFLD in CF patients.

Furthermore, these imaging features correlated well with ALP and GGT levels (*p* < 0.05) but not with the ALT and AST levels (*p* > 0.05), again underlining the cholestatic nature of CFLD. The lack of correlation of imaging findings with serum bilirubin levels (*p* > 0.05) is not surprising as bilirubin levels rise late in cholestatic liver diseases. This is, again, our explanation for the dissociation between the quantitative parameters, including liver volume, portal vein diameter and RLE. As for bilirubin, we would expect an increase in portal diameter or a decrease in RLE and liver volume only in very advanced CFLD. In early disease, the liver can still compensate. On the contrary, the splenic volume was the sole quantitative parameter that demonstrated significance (*p* < 0.05) [37]. We attribute this to either inflammatory/immunological changes or incipient, rather than significant, portal hypertension.

Although steatosis has been reported in up to 60% of CF patients [29] and is included in the Debray classification as one of the diagnostic criteria of CFLD, we found no significant hepatic fat fraction measured on CSI (*p* =0.30). This was expected, as there has been no proven relationship between steatosis and CFLD. When present, steatosis is likely a concomitant finding, either due to diabetes or medications [38]. Similarly, according to our results, hepatomegaly was not confirmed as a criterion of CFLD, which was not surprising since hepatomegaly as judged by clinical examination is known to be imprecise.

In general, CFLD is felt to be an emerging entity, Koh C. et al recently emphasized [39] the lack of sufficient characterisation and diagnostic tools for the diagnosis of adult-onset CFLD. They found that only 22% of CF patients had CFLD, based upon the Debray criteria, which they felt was likely an underestimation of actual disease.

Beyond finding an excellent sensitivity of MRCP [40] for depicting CFLD, we observed that none of the three imaging features (i.e. GBAM, PPT and PPFD) required CM administration for detection. However, gadoxetic acid-enhanced MRI simply improved detection of bile duct abnormalities and allowed us to evaluate liver function impairment based upon liver parenchymal enhancement in the hepatobiliary phase, i.e. 20 min after the injection of CM [41, 42].

The ALBI score associated well with the biomarkers of liver function derived from the uni- and multivariate analyses with regard to the severity of the disease, whereas the Child-Pugh score was associated only with the morphological changes including periportal fibrosis, bile duct abnormalities and liver contour irregularity, but not with contrast media uptake in the hepatobiliary phase. This is in keeping with the subjective nature of the CPS. The presence of altered liver morphology and decreased uptake of CM in the HBP were found in patients with more advanced disease, i.e. CPS B or ALBI grade 2. These findings can be explained by the fact that the ALBI score seems to be more sensitive in estimating disease severity than CPS, a fact already recognized in the literature [43, 44].

Our study’s limitations include, firstly, the retrospective nature of the majority of the cohort with the inherent limitations of a retrospective study. However, the results derived from this retrospective group were confirmed in the prospective group, which validated the applicability of the established classification algorithm. Another potential bias is that most of the patients had undergone lung transplantation and were therefore on immunosuppressants and other drugs, which are potential confounders of liver function results. However, our results were well in line with other clinical publications [7]. Furthermore, although our cohorts were not subject to histopathology staging, clinical, laboratory and MR features strongly suggest that the majority had early-stage CFLD. The lack of elevated serum bilirubin, as well as the absence of impaired uptake or excretion on hepatobiliary imaging support this conclusion [45].

There is no gold standard for CFLD diagnosis; even the universally-accepted Debray criteria, which may appear simpler and cheaper than liver MRI, is based largely on clinical signs, laboratory tests and ultrasound. Therefore, we feel that the integration of MRI in CFLD screening and follow-up might improve diagnosis of CFLD, when laboratory tests and/or clinical signs fail to do so.

In conclusion, a short unenhanced MRI protocol can identify the three cardinal imaging features of CFLD, namely altered gallbladder morphology, periportal tracking and periportal fat deposition. The hepatobiliary phase of gadoxetic acid-enhanced MRI can define the progression of CFLD.

## Abbreviations

3-T: 3-Tesla
ALBI: Albumin-bilirubin score
ALT: Alanine aminotransferase
ALP: Alkaline phosphatase
AP: Antero-posterior
AST: Aspartate aminotransferase
BDA: Bile duct abnormalities
CC: Craniocaudal
CF: Cystic fibrosis
CFLD: Cystic fibrosis-associated liver disease
CFTR: Cystic fibrosis transmembrane conductance regulator
CHAID: Chi-squared automatic interaction detector
CM: Contrast media
CPS: Child-Pugh score
CSI: Chemical shift imaging
CT: Computed tomography
DWI: Diffusion-weighted imaging
GBAM: Altered gallbladder morphology
Gd-EOB-DTPA: Gadolinium ethoxybenzyl diethylenetriaminepentaacetic acid
GGT: Gamma-glutamyltransferase
HBP: Hepatobiliary phase
LL: Latero-lateral
MRCP: Magnetic resonance cholangiopancreaticography
MRI: Magnetic resonance imaging
NPV: Negative predictive value
PACS: Picture Archiving and Communication System
PPF: Periportal fibrosis
PPFD: Periportal fat deposition
PPT: Periportal tracking
PPV: Positive predictive value
PSC: Primary sclerosing cholangitis
pv: Portal venous
RLE: Relative liver enhancement
RNH: Regenerative nodular hyperplasia
ROC: Receiver-operating characteristic
SI: Signal intensity
UDCA: Ursodeoxycholic acid
US: Ultrasound
